# The swine acute diarrhea syndrome coronavirus spike protein promotes syncytial formation via upregulation of cellular cholesterol synthesis

**DOI:** 10.1128/mbio.00976-25

**Published:** 2025-06-30

**Authors:** Dakai Liu, Miaomiao Zeng, Jiyu Zhang, Liaoyuan Zhang, Hongyan Shi, Xin Zhang, Jialin Zhang, Jianfei Chen, Zhaoyang Ji, Xiuwen Li, Gengting Gu, Tingshuai Feng, Da Shi, Dongbo Sun, Li Feng

**Affiliations:** 1 State Key Laboratory for Animal Disease Control and Prevention, Chinese Academy of Agricultural Sciences, Harbin Veterinary Research Institute 111613 , Harbin, China; 2 College of Animal Science and Veterinary Medicine, Heilongjiang Bayi Agricultural University 91625, Daqing, Heilongjiang, China; Cornell University College of Veterinary Medicine, Ithaca, New York, USA

**Keywords:** swine acute diarrhea syndrome coronavirus, cholesterol, spike protein, syncytia

## Abstract

Swine acute diarrhea syndrome coronavirus (SADS-CoV) is a novel coronavirus that causes acute diarrhea, vomiting, and high mortality in suckling piglets. Research has demonstrated that certain viruses enhance their replication by modulating intracellular cholesterol metabolism. However, the impact of SADS-CoV infection on cellular cholesterol synthesis remains unclear. Here, we found that SADS-CoV Spike (S) protein promoted syncytium formation by positively regulating cholesterol synthesis. Specifically, the virus upregulated the rate-limiting enzyme 3-hydroxy-3-methyl-glutaryl-CoA reductase through the inhibition of AMP-activated protein kinase (AMPK) activity. This inhibition was mediated by the activation of AKT-dependent phosphorylation of AMPKα at Ser485. Further investigation revealed that SADS-CoV S protein activated the PI3K/AKT pathway to promote cholesterol synthesis, a process that required the membrane protein integrin β1 (ITGB1). Importantly, we discovered that cholesterol facilitated cell-to-cell fusion mediated by the viral S protein, which enhanced syncytium formation. In summary, our findings demonstrate that the SADS-CoV S protein enhances cellular cholesterol accumulation by activating the PI3K/AKT/AMPK pathway through ITGB1, and that cholesterol facilitates syncytium formation mediated by the viral S protein. These insights contribute to a better understanding of SADS-CoV infection mechanisms and may inform future therapeutic strategies.

**IMPORTANCE:**

Cholesterol, a vital component of cellular membranes, is crucial for maintaining cell structure and function. It also acts as an essential host factor for the entry, replication, and propagation of various viruses. In this study, we show that the Spike protein of swine acute diarrhea syndrome coronavirus (SADS-CoV) promotes syncytial formation by upregulating cellular cholesterol synthesis. The viral Spike protein activates the PI3K/AKT signaling pathway, leading to increased cholesterol production through the inhibition of AMP-activated protein kinase (AMPK). This upregulation of cholesterol facilitates cell-to-cell fusion, a process that enhances viral spread and pathogenesis. Moreover, we demonstrate that integrin β1 (ITGB1) acts as a critical host factor that links the viral Spike protein to the activation of the PI3K/AKT pathway. ITGB1 interacts with the S protein, playing a pivotal role in viral replication and cholesterol synthesis regulation. Our findings highlight the critical role of cholesterol in SADS-CoV infection and provide a deeper understanding of the molecular mechanisms behind viral replication. This research opens up potential therapeutic strategies targeting cholesterol metabolism to mitigate the effects of SADS-CoV and similar viral infections.

## INTRODUCTION

Swine acute diarrhea syndrome coronavirus (SADS-CoV), also recognized as porcine/swine enteric alphacoronavirus, is a novel porcine coronavirus that has continued to infect suckling piglets across China since 2017 (1, 2). Characterized by acute diarrhea, vomiting, and high mortality rates, SADS-CoV shares clinical similarities with other porcine enteric coronaviruses (1, 2). SADS-CoV has four structural proteins: spike (S), membrane, nucleocapsid, and envelope, and the S protein is crucial in the early stages of viral infection (2–4). SADS-CoV exhibits a broad tropism for cells (5, 6), potentially due to the adaptability of the S protein to engage with host cell receptors. The significant cross-species transmission potential of SADS-CoV raises concerns about possible threats to the health of humans and other animals (7).

As a crucial component of mammalian cell membranes, cholesterol is not only essential for cellular functionality, but also plays a pivotal role in the pathogenicity of various viruses (8–11). Viral infections can induce changes to the activities of enzymes involved in cholesterol metabolism in host cells. For instance, hepatitis C virus (HCV) (12), avian influenza virus (13), dengue virus (DENV) (14), and porcine reproductive and respiratory syndrome virus (PRRSV) (15) regulate cholesterol synthesis by targeting 3-hydroxy-3-methyl-glutaryl-CoA reductase (HMGCR), the rate-limiting enzyme in cholesterol synthesis (16). Moreover, regulating the cholesterol content of the host cell membrane and interfering with intracellular cholesterol metabolism can effectively inhibit viral entry, fusion, translation, and replication (10, 11). However, the specific function of cholesterol metabolism in SADS-CoV infection remains unexplored. Hence, clarification of the involvement of host cholesterol in SADS-CoV infections offers a potential strategy to elucidate the mechanisms underlying viral replication.

The S protein of coronaviruses undergoes conformational changes upon viral entry, which are essential for facilitating membrane fusion (17, 18). In addition to virion-mediated fusion, the S protein on the plasma membrane can also induce receptor-dependent syncytium formation (19). Syncytia are multinucleated cells formed through the fusion of neighboring host cells, a process that facilitates the dissemination of the virus within the host. Furthermore, syncytium formation promotes viral replication by enabling direct cytoplasmic exchange between infected and non-infected cells (20). As a member of the coronavirus family, SADS-CoV-induced syncytia also depend on membrane fusion mediated by the S protein. However, the specific function of syncytium formation during SADS-CoV infection remains unclear. One notable mechanism in this process involves cholesterol, which contributes to syncytium formation mediated by the S protein of coronaviruses, including SARS-CoV-2 (19, 21, 22), severe acute respiratory syndrome coronavirus (23), and murine coronavirus (24).

Therefore, the aim of this study was to investigate the role of cholesterol in SADS-CoV replication. The results demonstrated that SADS-CoV enhances cholesterol biosynthesis via activating HMGCR and inhibition of AMP-activated protein kinase (AMPK) activity, which involved AKT-dependent phosphorylation of AMPKα at Ser485 mediated by SADS-CoV. Specifically, the viral S protein regulated cholesterol synthesis by activating the PI3K/AKT pathway through integrin β1 (ITGB1). Also, cholesterol promoted membrane fusion mediated by the SADS-CoV S protein, leading to syncytium formation, demonstrating that host cholesterol synthesis enhanced SADS-CoV infection, thereby providing new insights into the mechanism of coronavirus infection.

## MATERIALS AND METHODS

### Cells and viruses

African green monkey kidney (Vero E6), human embryonic kidney (HEK293T), porcine intestinal epithelial (IPI-2I), and human cervical carcinoma cells (HeLa) were cultured in Dulbecco’s modified Eagle’s medium (DMEM; 11965092; Life Technologies, Carlsbad, CA, USA). All media were supplemented with 10% fetal bovine serum (10099141C; Gibco, Life Technologies) at 37°C in a 5% CO_2_ environment. SADS-CoV (GenBank accession no. MF094681) is described previously (25).

### Antibodies (Abs) and reagents

SADS-CoV N protein-specific monoclonal antibody (mAb) 3E9 was prepared and maintained in our laboratory (26). Abs against Flag (ab205606), HA (ab9110), mouse IgG_1_ (ab190481), AMPK alpha 1 (ab32047), PI3K p85 alpha (ab191606), and AKT1 (ab227385) were sourced from Abcam (Cambridge, MA, USA). Cholesterol-water soluble (C4951), Methyl-β-cyclodextrin (MβCD; C4555), Filipin III solution (SAE0087), and Abs against Myc (M4439), HA (H9658), Flag (F1804), and GAPDH (G9545) were purchased from Sigma-Aldrich (St. Louis, MO, USA). Abs against phosphorylated (p)-AKT (Ser473) (4058T), p-AMPK alpha (Thr172) (2535S), and p-AMPK alpha (Ser485) (2537S) were obtained from Cell Signaling Technology (Danvers, MA, USA). Abs against PP2A alpha (A6702), HMGCR (A14741), and p-PP2A alpha (AP1043) were from ABclonal (Wuhan, China). An Ab against p-HMGCR (TA3865S) was acquired from Abmart (Shanghai, China). An Ab against ITGB1 (A-4) (sc-374429) was obtained from Santa Cruz Biotechnology (Shanghai, China). AICAR (HY-13417), AKT inhibitor VIII (HY-13055), Recilisib (HY-101625), LY294002 (HY-10108), Hoechst 33342 (R37605), and propidium iodide (PI, P4864) were purchased from MedChemExpress (Edison, NJ, USA). IRDye 800CW goat anti-mouse lgG secondary antibody (926-32210) and IRDye 680RD goat anti-rabbit lgG secondary Ab (925-68071) were from LiCor BioSciences (Lincoln, NE, USA). Alexa Fluor 488 goat anti-mouse IgG (H + L), Alexa Fluor 594 goat anti-mose IgG (H + L), and 40,6-diamidino-2-phenylindole (DAPI, R37606) were purchased from ThermoFisher Scientific (Waltham, MA, USA).

### Plasmid construction and transfection

The pCAGGS-Myc-SADS-CoV-S plasmid used in this study is described in a previous research (27). Similarly, the S1 subunit sequence (aa 1–545) of the S protein was engineered into the pCAGGS-Myc vector. DNA encoding the human PI3K p85α and p85β (GenBank accession numbers: NM_181523.3 and NM_005027.3), each containing an HA tag, was cloned into a pCAGGS vector. Additionally, DNA encoding human ITGB1 (GenBank accession number: NM_002211) was cloned into the pCAGGS-Flag vector. All of the plasmids described, including pEGFP-C1, were deposited in our laboratory. Vero E6, HEK293T, and IPI-2I cells were transfected with the above plasmids using X-treme GENE HP transfection reagent (06366546001; Roche, Switzerland).

### Immunofluorescence assay and confocal microscopy

IPI-2I and Vero E6 cells were cultured in 35 mm dishes. After infection with SADS-CoV or transfection with plasmids, cells were fixed with precooled 4% paraformaldehyde (16005, Sigma-Aldrich) in phosphate-buffered saline (PBS) for 30 min. They were then permeabilized with 0.1% Triton X-100 (T8787, Sigma-Aldrich) and 5% non-fat milk for 1 h. Cells were incubated with primary Abs for 6–8 h at 37°C, followed by three washes with PBS. Secondary Abs (Alexa 488 or 594-conjugated) or Filipin III were applied for 1 h, and nuclear DNA was stained with DAPI or PI for 15 min. After three washes with PBS, cells were observed using a LSM880-ZEISS confocal laser scanning microscope with Fast Airyscan (Zeiss, Germany).

### Animal experiment and immunohistochemistry (IHC) assay

Six 3-day-old specific pathogen-free piglets were randomly assigned to the experimental group (orally challenged with 5 × 10^4^ 50% tissue culture infectious dose [TCID_50_] of SADS-CoV) or an uninfected control group (oral challenged with DMEM). Post-inoculation, the piglets were monitored thrice daily for clinical symptoms, including vomiting, diarrhea, lethargy, and changes in body condition. The study concluded with euthanasia of all piglets 48 h post-infection (hpi), in accordance with the Ethical Committee guidelines. Representative sections of the small intestine tissue samples (duodenum, jejunum, and ileum) were fixed in 4% paraformaldehyde and stored in 70% ethanol at 4°C. IHC was performed as previously described (27). Slides were incubated overnight at 4°C with mAb 3E9 (1:50) against the SADS-CoV N protein, followed by incubation with horseradish peroxidase-labeled goat anti-mouse IgG_1_ for 1 h. Immunocomplexes were detected using the 3,3ʹ-diaminobenzidine liquid substrate system.

### Extraction and detection of tissue cell cholesterol

The intestinal tissue (0.1 g) from both SADS-CoV-infected and non-infected piglets is ground in liquid nitrogen and resuspended in 1 mL of isopropanol. The mixture is then centrifuged at 12,000 rpm for 10 min, and the supernatant is collected. Cholesterol content in the supernatant is measured using a total cholesterol (TC) detection kit (C1985; Solarbio Science and Technology Co., Ltd., Beijing, China). Absorbance is recorded at 500_nm_ using a multifunctional microplate reader (Envision, PerkinElmer, Waltham, MA, USA), and results are analyzed according to the manufacturer’s instructions.

### Western blot and co-immunoprecipitation (co-IP) assay

Western blot was performed as described previously (25). Briefly, cells were lysed with radio-immunoprecipitation assay buffer (R0278, Sigma-Aldrich) and centrifuged. The proteins were separated by electrophoresis with 12.5% sodium dodecyl sulfate-polyacrylamide gels, then electroblotted onto nitrocellulose membranes (66485, Pall Corporation, Port Washington, NY, USA) which were blocked with 5% non-fat milk and incubated overnight at 4°C with primary Abs, followed by IRDye 800CW or IRDye 680RD secondary Abs for 45 min in the dark. Blots were visualized with an Odyssey infrared imaging system (LiCor BioSciences). For Co-IP assays, cells were lysed in IP buffer (87788, ThermoFisher Scientific) with phenylmethanesulfonyl fluoride (ST506-2, Beyotime Biotechnology, Shanghai, China) and protease inhibitors (04693132001, Roche, Switzerland), centrifuged, and incubated with primary Abs overnight. The lysates were then incubated with protein A/G magnetic beads (88803, ThermoFisher Scientific), washed, and analyzed by western blot.

### Drug treatment assay and cell viability assay

In accordance with the experimental requirements, IPI-2I and Vero E6 cells were pretreated with various drugs or dimethyl sulfoxide (HY-Y0320, MedChemExpress, USA) before inoculation with SADS-CoV (multiplicity of infection [MOI] = 0.1]. After incubation for 1 h, the supernatants were replaced with fresh media containing chemicals, and the cells were incubated at 37°C until sample collection. For cell viability assessment, IPI-2I and Vero E6 cells in 96-well plates were treated with Abs or drugs for 24 h. Cell viability was measured using Cell Counting Kit-8 (CCK-8; CK04, Dojindo Laboratories Co., Ltd., Kumamoto, Japan) according to the manufacturer’s instructions. The cytotoxic concentration of 50% (CC_50_) of the compounds was calculated using GraphPad Prism 8.0 software (GraphPad Software, LLC, San Diego, CA, USA).

### RNA interference

IPI-2I and Vero E6 cells were seeded in the wells of 12-well plates, grown to 50% confluency, and then transfected for 48 h with 50 nM small interfering RNA (siRNA) targeting the human and swine ITGB1 gene sequence (5ʹ→3ʹ) GAACAGATCTGATGAATGAAA (Ruibo Biotechnology Co., Ltd., Guangdong, China) using Lipofectamine RNAiMAX Reagent (13778075; ThermoFisher Scientific). Following transfection, the cells were infected with SADS-CoV (MOI = 0.1), and samples were collected 36 hpi.

### Ab blocking assay

IPI-2I and Vero E6 cells were seeded in the wells of 24-well plates. The medium was then replaced with fresh DMEM containing 20 µg/mL ITGB1 Ab or the same concentration of mouse IgG_1_ Ab as a control. After 1 h of incubation at 4°C, the cells were washed with chilled PBS and infected with SADS-CoV (MOI = 0.1) in the presence of 5 µg/mL of trypsin without ethylenediaminetetraacetic acid (EDTA) for 1 h at 4°C. Following three washes, medium with 20 µg/mL ITGB1 or IgG_1_ Ab and 5 µg/mL of trypsin (without EDTA) was added, and the cells were incubated at 37°C for 36 h.

### TCID_50_ assay

Vero E6 cells were cultured in the 96-well plates to 90% confluency and infected with 10-fold graded dilutions of the virus. The medium was removed after the cells were incubated with viruses for 1 h at 37°C. The cells were incubated with DMEM containing 5 µg/mL of trypsin (without EDTA). Cytopathic effects were observed under an inverted microscope 3–5 days post-infection, and virus titers were determined using the Reed–Muench method.

### RNA extraction, reverse transcription, and qRT-PCR

Total RNA was extracted from cells using the Simply P Total RNA Extraction Kit (BSC52S1; BioFlux, Brussels, Belgium). The extracted RNA (1 µg) was reverse-transcribed into complementary DNA (cDNA) using the PrimeScript IV First Strand cDNA Synthesis Mix (6215A; TaKaRa, Japan). Then, qRT-PCR analysis was conducted on a QuantStudio 5 system (Applied Biosystems, Foster City, CA, USA) with TB Green Premix Ex Taq II (RR820A; TaKaRa, Japan) and the primers provided in Table S1. Relative mRNA levels were analyzed using the 2^−∆∆Ct^ method.

### Host factor screening for SADS-CoV infection

The Silencer Select human cell surface siRNA library (A30144, ThermoFisher Scientific) contains 2,382 siRNAs targeting 794 membrane protein genes. In accordance with the manufacturer’s instructions, HeLa cells were transfected using this library with Lipofectamine RNAi MAX transfection reagent, applying a concentration of 50 µM for the specific target siRNA, while using an equal concentration of siNC as a negative control. After 48 hours post-transfection (hpt), the cells were infected with SADS-CoV (MOI = 0.1) for 24 h. The cells were then fixed with 4% paraformaldehyde and blocked with 5% non-fat dry milk. Subsequently, a mouse mAb 3E9 targeting the SADS-CoV N protein was added and incubated overnight at 4°C. After washing, the cells were incubated in the dark for 45 min with a fluorescently labeled secondary antibody [Alexa Fluor 488 goat anti-mouse IgG (H + L)]. Nuclei were stained with DAPI for 15 min. Finally, the cells were observed using an inverted fluorescence microscope (EVOS M5000, Life, USA). Genes that inhibited SADS-CoV infection by over 80% were selected to identify potential host factors influencing viral infection.

### Membrane fusion assay

Vero E6 cells were seeded in the wells of 12-well plates and cultured to 70% confluence. Plasmids expressing the SADS-CoV S protein and/or EGFP were transfected into the cells. At 6 hpt, the medium was refreshed, and cholesterol and drugs were added as needed. At 12 hpt, the cells were treated with 5 µg/mL of trypsin (without EDTA). The cells were then fixed and stained with Hoechst 33342 for 15 min at 36 hpt and imaged using an inverted fluorescence microscope (EVOS M5000, Life). Quantification of membrane fusion induced by SADS-CoV S protein was performed by calculating the number of cells in GFP^+^ syncytia.

### Statistical analysis

All results shown in the figures were presented, where appropriate, as mean and standard deviation (SD). The gray level of protein bands and the fluorescence intensity were analyzed with ImageJ software. The *t*-test was conducted, and bar charts were generated using GraphPad Prism 8.0 software. A probability (*P*) value of <0.05, as determined with the two-tailed unpaired Student’s *t*-test, was considered statistically significant. “ns” indicates no significant difference.

## RESULTS

### SADS-CoV infection enhances cellular cholesterol accumulation

**Fig 1:**
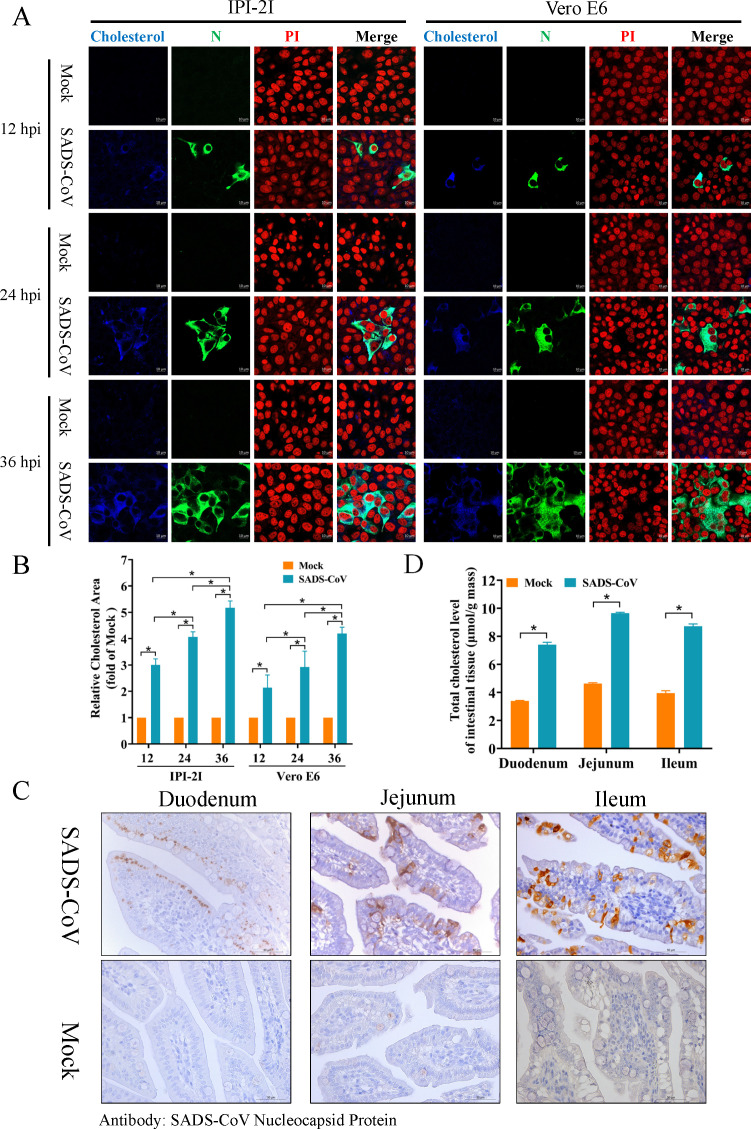
SADS-CoV infection enhances cellular cholesterol accumulation. (**A**) Detection of cellular cholesterol levels following SADS-CoV infection. IPI-2I and Vero E6 cells were infected with SADS-CoV (MOI = 0.1) and fixed at 12, 24, and 36 hpi for immunofluorescence analysis. Fliplin was employed to stain intracellular cholesterol (blue), while SADS-CoV N proteins were counterstained with mAb 3E9 (green). Cell nuclei were labeled with PI (red). Scale bar = 10 µm. (**B**) Cholesterol levels were quantified by measuring the blue fluorescence levels in SADS-CoV-infected cells compared to mock-infected cells using ImageJ software. (**C**) Representative microphotographs of viral antigen immunochemical staining in SADS-CoV-infected and non-infected small intestine tissues (duodenum, jejunum, and ileum) are presented. The virus in intestinal tissue cells was immunostained using the mAb 3E9 against the SADS-CoV N protein. Scale bars = 50 µm. (**D**) The cholesterol of piglet small intestinal tissue (0.1 g) was determined using a total cholesterol detection kit. Absorbance was recorded at 500_nm_ with a multifunctional microplate reader, and the total cholesterol in the small intestinal tissue was calculated based on a standard curve. The results are reported as the means and SD (error bars) of three independent experiments (*P < 0.05).

To examine the impact of SADS-CoV infection on cellular cholesterol levels, IPI-2I and Vero E6 cells were inoculated with SADS-CoV (MOI = 0.1) and fixed at 12, 24, and 36 hpi for immunofluorescence analysis. Filipin staining was employed to observe changes in intracellular cholesterol distribution. Notably, SADS-CoV-infected cells showed a marked increase in blue fluorescence intensity, indicating higher cholesterol levels, as compared to uninfected controls (Fig. 1A). Additionally, quantitative analysis of blue fluorescence intensity further substantiated the observation of heightened cholesterol accumulation in both IPI-2I and Vero E6 cells following SADS-CoV infection (Fig. 1B). To validate these *in vitro* findings, TC levels were measured in small intestinal tissues from piglets infected with SADS-CoV. IHC confirmed that SADS-CoV successfully infected cells in the duodenum, jejunum, and ileum of piglets (Fig. 1C). Moreover, TC levels in the intestinal segments were significantly higher in SADS-CoV-infected piglets compared to the control group (Fig. 1D). Collectively, findings from both *in vitro* and *in vivo* experiments underscore that SADS-CoV infection enhances cellular cholesterol accumulation.

### Increased activity of HMGCR in SADS-CoV-infected cells is mediated by AMPK inhibition

**Fig 2:**
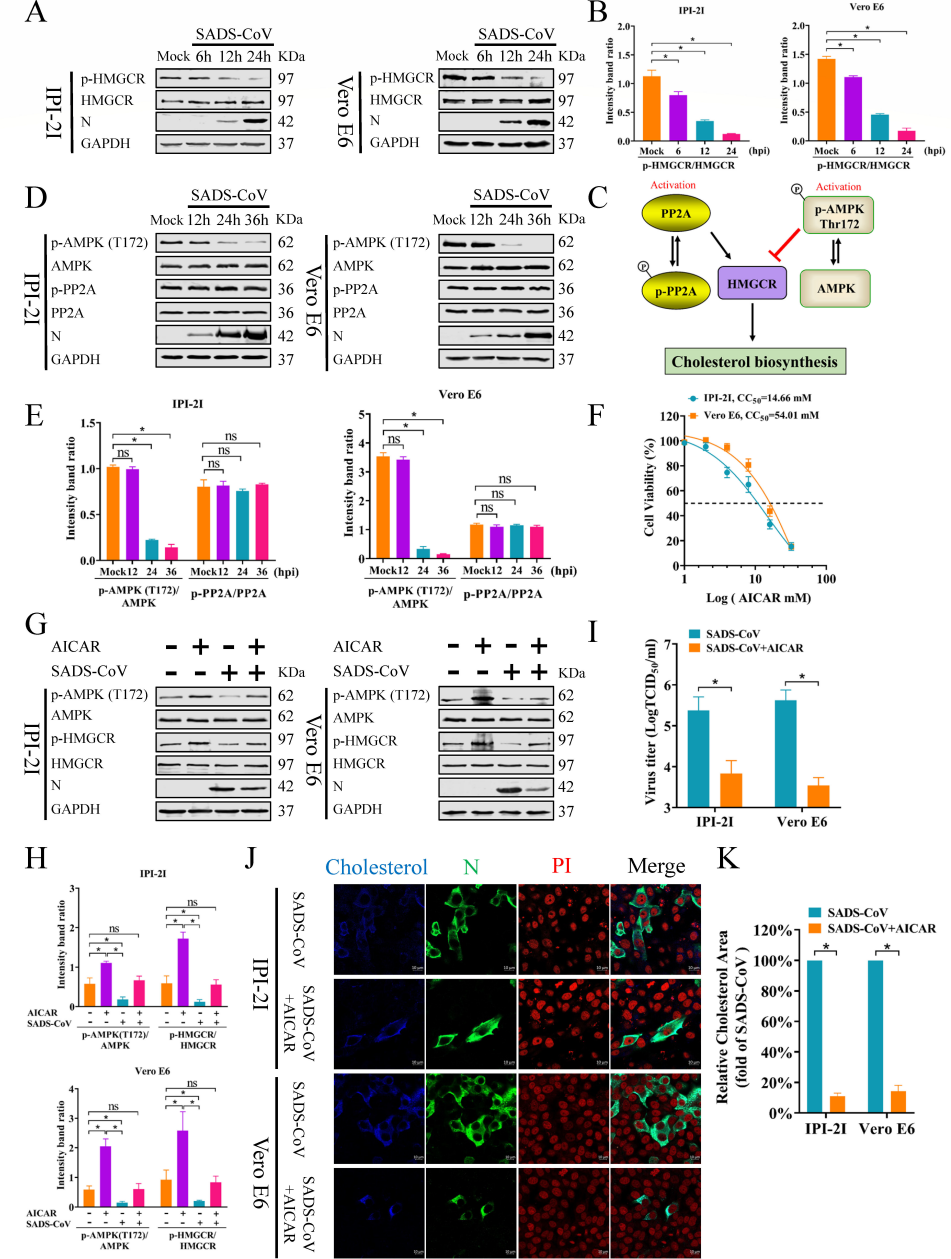
Increased activity of HMGCR in SADS-CoV-infected cells is mediated by AMPK inhibition. (**A, B**) IPI-2I and Vero E6 cells infected with SADS-CoV (MOI = 0.1) were harvested at 6, 12, and 24 hpi. Cellular lysates were used for western blot with Abs against p-HMGCR, HMGCR, and the SADS-CoV N protein. GAPDH was used as a protein loading control (**A**). Relative intensity of p-HMGCR was normalized to the total HMGCR using gray value analysis (**B**). (**C**) A model of cholesterol synthesis showing that AMPK and PP2A regulate the HMGCR pathway. AMPK inhibits HMGCR activity through phosphorylation, thereby reducing cholesterol synthesis, while PP2A enhances HMGCR activity through dephosphorylation, promoting cholesterol synthesis. (**D, E**) IPI-2I and Vero E6 cells infected with SADS-CoV (MOI = 0.1) were harvested at 12, 24, and 36 hpi. Cellular lysates are used for western blot with Abs against p-AMPK (T172), total AMPK, p-PP2A, total PP2A, SADS-CoV N protein, and GAPDH (**D**). Relative intensities of p-AMPK (T172) normalized to total AMPK and p-PP2A normalized to total PP2A were quantified using grayscale analysis (**E**). (**F**) Cytotoxic effect of AICAR on IPI-2I and Vero E6 cells. (G–I) IPI-2I and Vero E6 cells were either mock-treated or pretreated with 2 mM AICAR for 1 h, followed by SADS-CoV exposure for 1 h. Cells were then cultured with or without 2 mM AICAR for 36 h. Western blots were performed using Abs against p-AMPK (T172), AMPK, p-HMGCR, HMGCR, SADS-CoV N protein, and GAPDH (**G**). Relative intensities of p-AMPK (T172) normalized to total AMPK and p-HMGCR normalized to total HMGCR were quantified using grayscale analysis (**H**). Viral titers were determined using TCID_50_ assays in Vero E6 cells at 36 hpi. (**I**). (**J, K**) Cells are pretreated and infected as described in G, cells were fixed at 36 hpi and examined by immunofluorescence. Cholesterol stained with Filipin (blue), SADS-CoV N protein co-stained with mAb 3E9 (green). Cell nuclei labeled with PI (red). Scale bar = 10 µm (**J**). Blue fluorescence levels of SADS-CoV-infected and mock-infected cells were measured using ImageJ software to quantify cholesterol levels (**K**). The results are reported as the means and SD (error bars) of three independent experiments (*P < 0.05). ns, no significant difference.

To explore how SADS-CoV impacts cellular cholesterol synthesis, we measured the protein levels of both HMGCR and p-HMGCR, a key cholesterol biosynthesis enzyme that is inhibited by phosphorylation at serine 872 (16). IPI-2I and Vero E6 cells were infected with SADS-CoV (MOI = 0.1) and collected at 6, 12, and 24 hpi for western blot. As shown in Fig. 2A, p-HMGCR decreased over time during infection, while HMGCR levels remained unchanged. Further analysis of protein densitometry revealed a lower p-HMGCR/HMGCR ratio in infected cells compared to controls (Fig. 2B). These findings suggested that SADS-CoV infection upregulated HMGCR activity. Previous studies indicate that HMGCR phosphorylation is mainly regulated by the upstream effectors AMPK and PP2A (Fig. 2C) (28, 29). PP2A is a serine/threonine phosphatase that increases HMGCR activity and boosts cholesterol synthesis through dephosphorylation (29). Conversely, phosphorylation of AMPKα at Thr172 activates AMPK, and activated AMPK inhibits HMGCR activity through direct phosphorylation, thereby suppressing cholesterol synthesis (28, 30). Hence, the phosphorylation status of AMPK and PP2A was assessed in SADS-CoV-infected cells at different times. As shown in Fig. 2D and E, phosphorylation of AMPK at Thr172 was decreased in SADS-CoV-infected cells at 24 and 36 hpi, whereas the total AMPK protein remained unaltered. Conversely, neither PP2A nor p-PP2A exhibited apparent alterations. These results suggest that the enhanced HMGCR activity in SADS-CoV-infected cells was due to AMPK activity inhibition, not PP2A involvement.

To confirm these findings, the AMPK activator AICAR was used to stimulate AMPK activity and examine its effect on HMGCR activity and cholesterol levels in SADS-CoV-infected cells. The results showed that AICAR at 2 mM exhibited no cytotoxicity in IPI-2I and Vero E6 cells (Fig. 2F). Western blot revealed that the phosphorylation levels of AMPK at Thr172 and HMGCR were upregulated in AICAR-treated cells compared to non-treatment cells (Fig. 2G and H), indicating that the activation of AMPK inhibited HMGCR activity and cholesterol synthesis. Additionally, AICAR-treated cells exhibited reduced levels of SADS-CoV N protein (Fig. 2G) and lower viral titers (Fig. 2I), suggesting that AMPK activation also impedes viral replication. Then, immunofluorescence analysis to investigate the effect of AICAR on cellular cholesterol confirmed that AICAR decreased both cholesterol (blue) and viral protein levels (green) (Fig. 2J and K). These findings indicate that upregulation of AMPK activity reduced cholesterol synthesis and inhibited SADS-CoV replication. Collectively, these findings demonstrated that increased activity of HMGCR in SADS-CoV-infected cells was mediated by reduced AMPK activity, which prevented HMGCR phosphorylation and led to heightened HMGCR activity.

### Inhibition of AMPK activity is mediated by SADS-CoV via AKT phosphorylation of AMPK at Ser485

**Fig 3:**
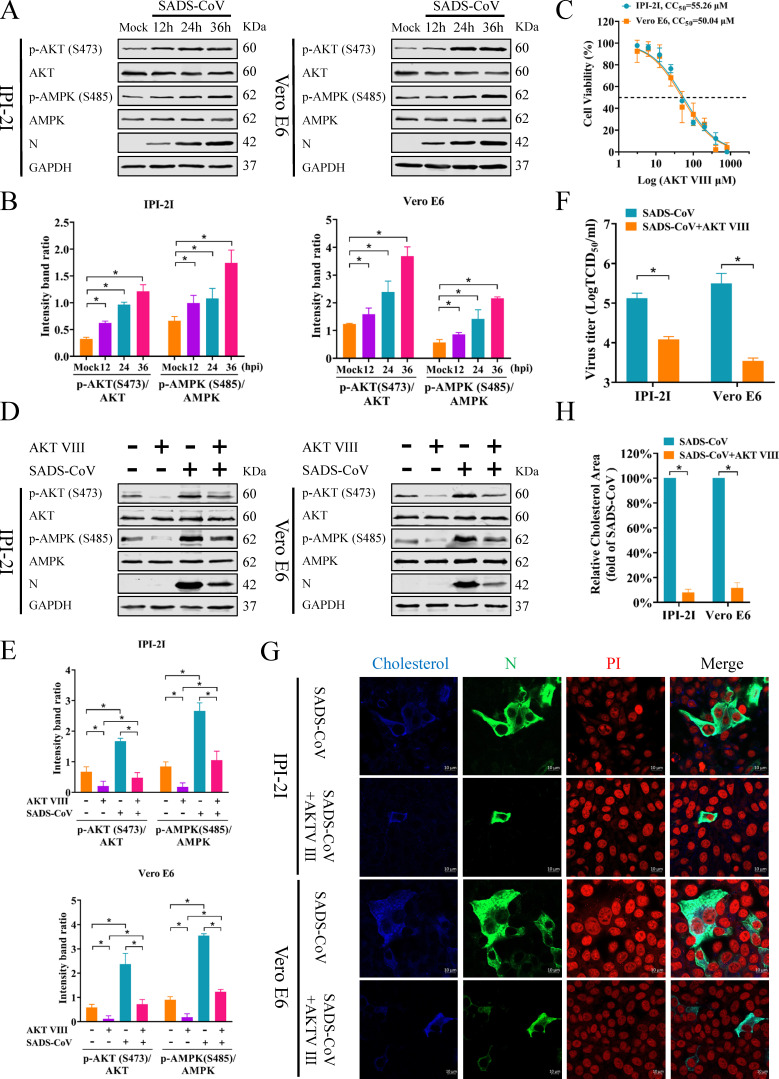
Inhibition of AMPK activity is mediated by SADS-CoV via AKT phosphorylation of AMPK at Ser485. (**A, B**) IPI-2I and Vero E6 cells infected with SADS-CoV (MOI = 0.1) were harvested at 12, 24, and 36 hpi. Western blots were performed using Abs against p-AKT (S473), AKT, p-AMPK (S485), AMPK, SADS-CoV N protein, and GAPDH (**A**). Relative intensities of p-AKT (S473) normalized to total AKT and p-AMPK (S485) normalized to total AMPK were quantified using grayscale analysis (**B**). (**C**) Cytotoxic effect of AKT VIII on IPI-2I and Vero E6 cells. (D–F) IPI-2I and Vero E6 cells were either mock-treated or pretreated with 12.5 µM AKT VIII for 1 h, followed by SADS-CoV exposure for 1 h, and then cultured with or without 12.5 µM AKT VIII for 36 h. Western blots were performed using Abs against p-AKT (S473), AKT, p-AMPK (S485), AMPK, SADS-CoV N protein, and GAPDH (**D**). Gray analysis of the relative expression intensities of p-AKT (S473) and p-AMPK (S485) as compared to total AKT and AMPK (**E**). Viral titers were determined using TCID_50_ assays (**F**). (**G, H**) Cells are pretreated and infected as described in (A), fixed at 36 hpi, and examined by immunofluorescence. Cholesterol stained with Filipin (blue), SADS-CoV N protein co-stained with mAb 3E9 (green). Cell nuclei labeled with PI (red). Scale bar = 10 µm (**G**). Blue fluorescence levels of SADS-CoV-infected and mock-infected cells were measured using ImageJ software to quantify cholesterol levels (**H**). The results are reported as the means and SD (error bars) of three independent experiments (*P < 0.05).

We next aimed to uncover the mechanism underlying the inhibition of AMPK by SADS-CoV. Previous studies indicate that AMPK activity is not only confined to the phosphorylation at Thr172, but regulated by the serine/threonine kinase AKT, which inhibits AMPK activity via phosphorylation of Ser485 (31–33). Specifically, AKT-induced phosphorylation of AMPK at Ser485 reduces Thr172 phosphorylation and consequently decreases AMPK activity. Notably, HCV suppresses AMPK activity by phosphorylating AKT at Ser473, which facilitates phosphorylation of AMPK at Ser485 (34). This mechanism may be employed by SADS-CoV. Therefore, the phosphorylation status of AMPK at Ser485 and AKT at Ser473 in SADS-CoV-infected cells was investigated. As shown in Fig. 3A and B, phosphorylation of AKT at Ser473 and AMPK at Ser485 was increased, suggesting that SADS-CoV suppressed AMPK activity by phosphorylating AMPK at Ser485 via AKT.

Our hypothesis stated that targeting AKT could effectively prevent inhibition of AMPK driven by AKT. So, we used AKT VIII, an AKT inhibitor, to restrain AKT activity and assess its effects on AMPK phosphorylation and cholesterol synthesis. The results of the cell viability assay indicated that AKT VIII showed no cytotoxicity at 12.5 µM (Fig. 3C). Western blot revealed that AKT VIII decreased p-AKT (Ser473) and p-AMPK (Ser485) levels, while total AKT and AMPK remained unchanged (Fig. 3D and E), indicating that AKT inhibition disrupts AMPK phosphorylation at Ser485 in SADS-CoV-infected cells. Additionally, AKT VIII reduced SADS-CoV N protein (Fig. 3D) and viral titers (Fig. 3F), indicating inhibition of viral replication. Next, the effect of AKT VIII on cellular cholesterol synthesis was assessed. The results showed that the blue fluorescence of AKT VIII-treated cells was decreased as compared with non-treated control cells (Fig. 3G and H), indicating a reduction in cholesterol synthesis. Furthermore, green fluorescence of the SADS-CoV N protein was obviously reduced in AKT VIII-treated cells (Fig. 3G). Taken together, these findings suggested that SADS-CoV inhibited AMPK activity via AKT-mediated phosphorylation of AMPK at Ser485.

### AKT activation by SADS-CoV is dependent on PI3K

**Fig 4:**
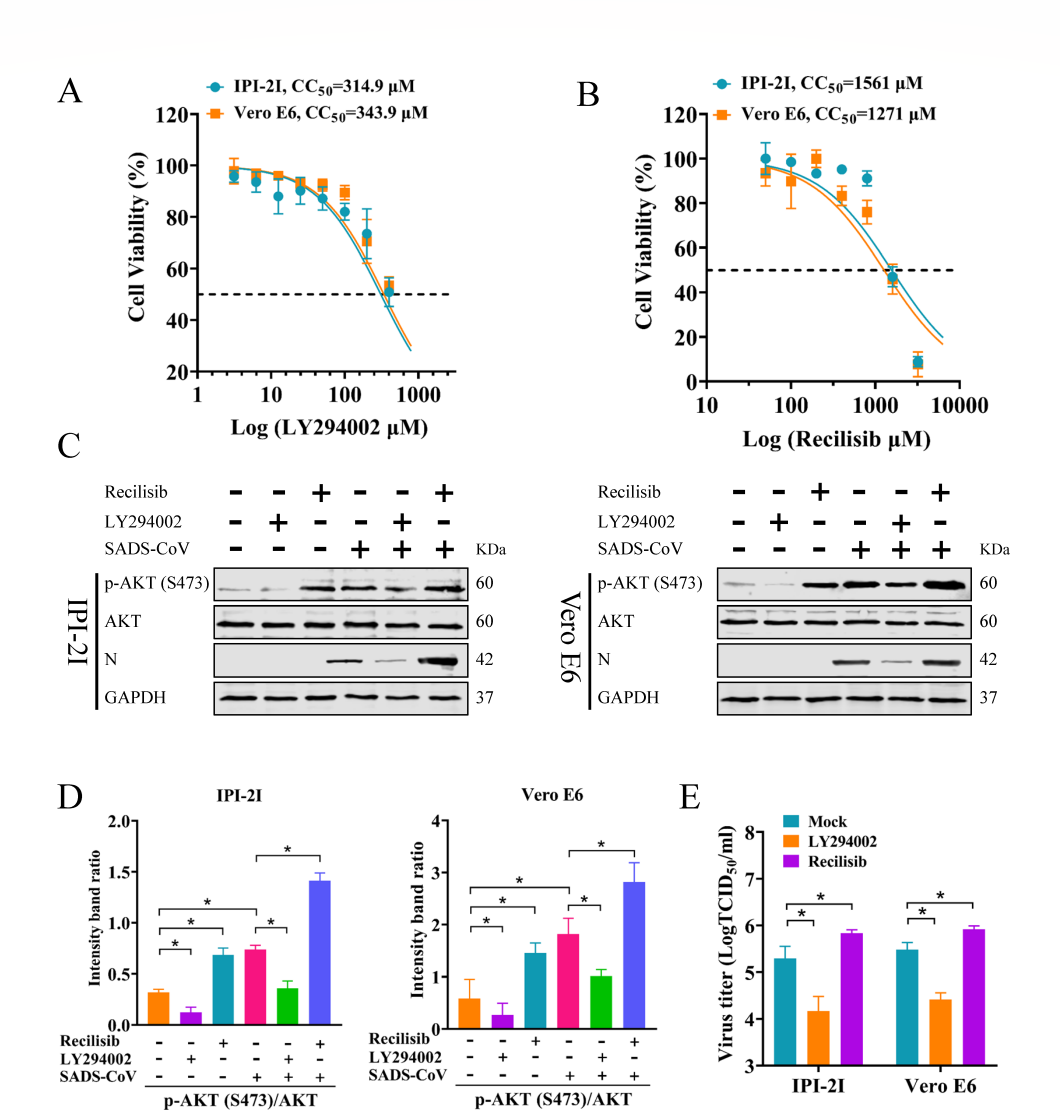
AKT activation by SADS-CoV is dependent on PI3K. (**A, B**) Cytotoxic effect of LY294002 and Recilisib on IPI-2I and Vero E6 cells. (C–E) IPI-2I and Vero E6 cells were either mock-treated or pretreated with LY294002 (100 µM) and Recilisib (800 µM) for 1 h, followed by SADS-CoV exposure for 1 h, and then cultured with or without LY294002 (100 µM) and Recilisib (800 µM) for 36 h. Western blots were performed using Abs against p-AKT (S473), AKT, SADS-CoV N protein, and GAPDH (**C**). Gray analysis of the relative expression intensities of p-AKT (S473) as compared to total AKT (**D**). Viral titers were determined using TCID_50_ assays (**E**). The results are reported as the means and SD (error bars) of three independent experiments (*P < 0.05).

AKT phosphorylation depends on PI3K, which recruits AKT to the cell membrane (35–37). Although a previous study has highlighted the critical role of the PI3K/AKT pathway in SADS-CoV infection through transcriptomic analysis and pathway enrichment with reference to the Kyoto Encyclopedia of Genes and Genomes (KEGG) (38), these conclusions were primarily based on gene expression data and did not directly validate changes at the protein level. To investigate the role of the PI3K/AKT pathway in regulating cellular cholesterol synthesis during SADS-CoV infection, the PI3K inhibitor LY294002 and PI3K activator Recilisib were utilized to assess the expression levels of AKT and p-AKT (Ser473) following SADS-CoV infection. The results of the cell viability assay indicated that neither compound was cytotoxic to IPI-2I and Vero E6 cells at 100 and 800 µM, respectively (Fig. 4A and B). Cells were pretreated with LY294002 or Recilisib, infected with SADS-CoV (MOI = 0.1), and collected at 36 hpi. Western blot revealed that LY294002 reduced phosphorylation of AKT at Ser473, while Recilisib had an opposite effect, with no obvious change in total AKT levels (Fig. 4C and D). Furthermore, SADS-CoV N protein levels decreased in LY294002-treated cells and increased in Recilisib-treated cells. Correspondingly, the TCID_50_ assay showed a decreased viral titer in LY294002-treated cells and an increased titer in Recilisib-treated cells (Fig. 4E). These findings demonstrate that SADS-CoV activated AKT in a PI3K-dependent manner, which was consistent with the predictions from transcriptomic analysis and provides direct experimental evidence supporting the role of this pathway in SADS-CoV infection.

### Cholesterol synthesis is promoted by the SADS-CoV S protein via the PI3K/ AKT pathway

**Fig 5:**
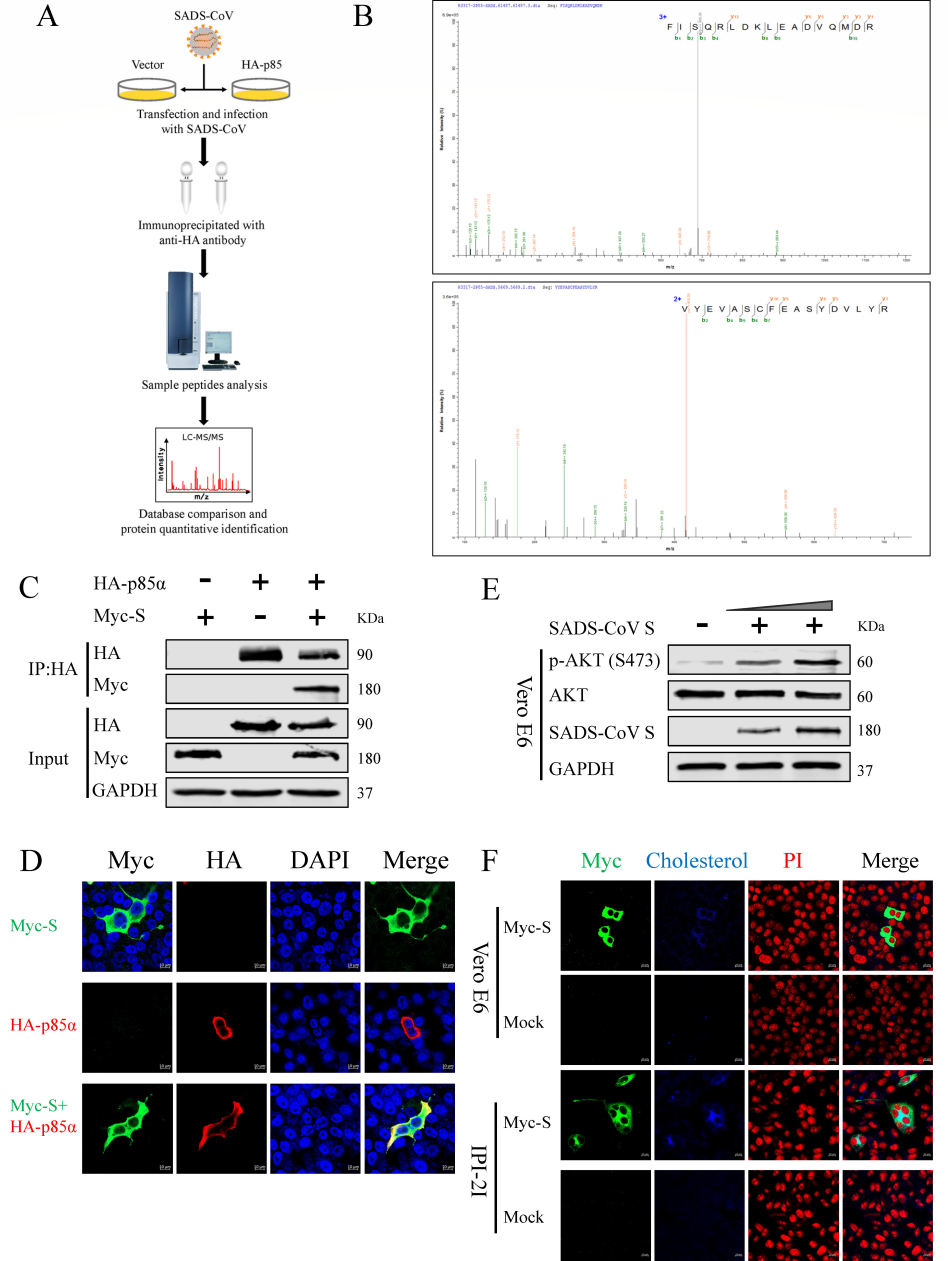
Cholesterol synthesis is promoted by SADS-CoV S protein through the PI3K/ AKT pathway. (**A**) SADS-CoV S protein was found to interact with PI3K p85α by LC-MS/MS. Vero E6 cells transfected with HA-tagged p85α, p85β, or control for 36 h, then infected with SADS-CoV (MOI = 0.1) for 24 h, followed by immunoprecipitation using an anti-HA Ab and analysis via LC-MS/MS. (**B**) LC-MS/MS spectra of peptides of SADS-CoV S protein. (**C**) Co-IP analysis of the interaction between SADS-CoV S protein and p85α. HEK293T cells co-transfected with plasmids for SADS-CoV S protein and p85α, incubated for 36 h, and collected for co-IP analysis using an anti-HA Ab. (**D**) Co-localization of SADS-CoV S protein and p85α. Vero E6 cells co-transfected with plasmids expressing SADS-CoV S protein (green) and p85α (red). Merged images show co-localization of these proteins. Nuclei are highlighted by DAPI staining (blue) in the merged images. (**E**) Vero E6 cells transfected with Myc-tagged SADS-CoV S plasmids (0, 1.5, and 3 µg). Western blots were performed to detect p-AKT (S473), AKT, SADS-CoV S protein, and GAPDH. (**F**) Overexpression of the SADS-CoV S protein promoted cholesterol synthesis. Vero E6 and IPI-2I cells were transfected with 3 µg of Myc-tagged SADS-CoV S plasmid or an empty vector and fixed at 24 hpt for immunofluorescence analysis. Intracellular cholesterol was stained with Fliplin (blue), SADS-CoV S proteins were detected using anti-Myc Ab (green), and cell nuclei labeled with PI (red).

PI3K consists of a regulatory subunit (p85, including α and β isoforms) and a catalytic subunit (p110, comprising α, β, γ, and δ isoforms), activated by autophosphorylated tyrosine kinase receptors, nonreceptor tyrosine kinases, or specific viral proteins (39, 40). Notably, the p85 subunit can also be activated through interactions with certain viral proteins (41–44). To investigate whether the proteins of SADS-CoV had a similar effect, Co-IP assays and liquid chromatography-tandem mass spectrometry (LC-MS/MS) were conducted to identify viral proteins interacting with p85 (Fig. 5A). Vero E6 cells were transfected with HA-tagged p85α and p85β plasmids and subsequently infected with SADS-CoV. All cell lysates were immunoprecipitated using an anti-HA Ab and analyzed by LC-MS/MS. Moreover, immunoprecipitated samples from cells transfected with the pCAGGS-HA plasmids were used as negative controls to eliminate nonspecific interactions. The LC-MS/MS analysis identified interactions between p85α and the SADS-CoV S protein and NS3 (Table S2), while p85β showed no interaction (data not shown). Given that the protein score (Table S2) and identified peptides (Fig. 5B) of the S protein are superior to those of NS3, the S protein was chosen for further experiments.

Co-IP analysis of HEK293T cells co-transfected with the Myc-SADS-CoV S plasmid and the HA-p85α plasmid confirmed an interaction between the PI3K regulatory subunit p85α and the S protein (Fig. 5C). Additionally, immunofluorescence analysis demonstrated co-localization of p85α and SADS-CoV S protein in the cytoplasm (Fig. 5D). These observations suggest a significant interaction between the SADS-CoV S protein and the PI3K regulatory subunit p85α. To further investigate the impact of the S protein on cellular cholesterol, overexpression analysis was performed, which revealed increased phosphorylation of AKT at Ser473 (Fig. 5E), indicating that the S protein stimulated the PI3K/AKT pathway. Furthermore, immunofluorescence analysis showed strong blue fluorescence in Vero E6 and IPI-2I cells overexpressing the S protein, whereas no obvious blue fluorescence was observed in the control group, suggesting that overexpression of the S protein promoted cellular cholesterol synthesis (Fig. 5F). Collectively, these findings suggested that the SADS-CoV S protein enhanced cellular cholesterol synthesis via the PI3K/AKT pathway.

### SADS-CoV induces cellular cholesterol synthesis via integrin β1

**Fig 6:**
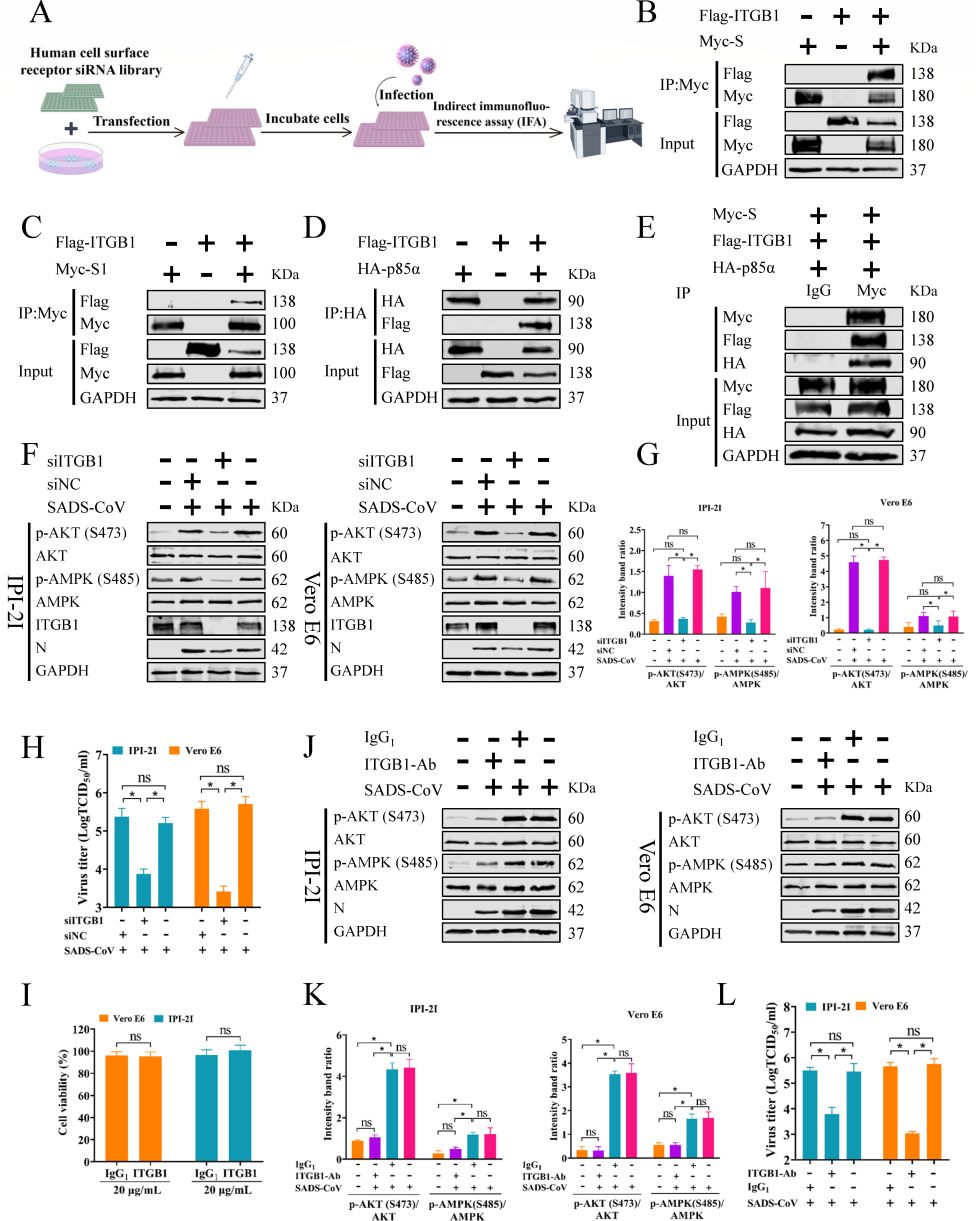
SADS-CoV induces cellular cholesterol synthesis via ITGB1. (**A**) Schematic diagram of the screening for membrane proteins influencing SADS-CoV infection. Immunofluorescence analysis identification of cell membrane proteins in HeLa cells influencing SADS-CoV infection using a Silencer Select human cell surface siRNA library. (**B, C**) Co-IP showing ITGB1 interaction with the S1 domain of the SADS-CoV S protein. HEK293T cells co-transfected with plasmids for SADS-CoV S or S1 and ITGB1, incubated for 36 h, and then collected for co-IP assay using an anti-Myc Ab. (**D**) Co-IP indicating the interaction between ITGB1 and the PI3K p85α. HEK293T cells co-transfected with plasmids for ITGB1 and p85α for 36 h, and then collected for co-IP assay using an anti-HA Ab. (**E**) Co-transfection revealed protein interactions between SADS-CoV S, ITGB1, and p85α. HEK293T cells were co-transfected with plasmids encoding SADS-CoV S protein, ITGB1, and p85α for 36 h, and then harvested for Co-IP assay using an anti-Myc Ab and control IgG. (F–H) siRNA knockdown reduced expression of ITGB1. Cells were treated with either siNC or siITGB1 (50 nM) for 48 h and then infected with SADS-CoV (MOI = 0.1) for 36 h. Western blots were performed using Abs against p-AKT (S473), AKT, p-AMPK (S485), AMPK, SADS-CoV N protein, and GAPDH (**F**). Gray analysis of the relative expression intensities of p-AKT (S473) and p-AMPK (S485) as compared to total AKT and AMPK (**G**). Viral titers were determined using TCID_50_ assays (**H**). (**I**) Cytotoxic effects of IgG_1_ and ITGB1 Abs on IPI-2I and Vero E6 cells. (J–L) Blocking with anti-ITGB1 Ab. IPI-2I and Vero E6 cells were pretreated with 20 µg/mL ITGB1 or control IgG_1_ Ab at 4°C for 1 h, and then infected with SADS-CoV (MOI = 0.1) for 1 h. After washing three times, the same Ab treatments continued, and cells were incubated at 37°C for 36 h before sample collection. Western blots were conducted with the indicated Abs above (**J**). Gray analysis of the comparative expression levels of p-AKT (S473) and p-AMPK (S485) relative to total AKT and AMPK, respectively (**K**). TCID_50_ assays were utilized to determine viral titers (**L**). The results are reported as the means and SD (error bars) of three independent experiments (*P < 0.05).

A recent study found that SADS-CoV induces autophagy through integrin α3, which might be mediated by the AKT/mTOR pathway (45). Although the mechanism utilized by SADS-CoV to regulate integrin α3-induced activation of the PI3K/AKT pathway remains unclear, the α3β1 integrin complex, formed by ITGA3 and integrin β1 (ITGB1), may be involved in this process. The results of the present study demonstrated the SADS-CoV S protein activated the PI3K/AKT pathway, but it is still unknown whether other proteins mediate this interaction. A Silencer Select human cell surface siRNA library was utilized to screen for host factors of SADS-CoV infection by interfering with membrane protein expression in HeLa cells. Following SADS-CoV infection, immunofluorescence analysis was conducted to identify membrane proteins affecting viral replication (Fig. 6A). The screening results (data not shown) found that ITGB1 may play an important role in SADS-CoV replication. In addition, ITGB1 has been reported to mediate vaccinia virus entry through the PI3K/AKT pathway (46). Based on our screening results and previous studies, ITGB1 may play a potential role between the SADS-CoV S protein and the PI3K/AKT pathway. So, Co-IP analysis was employed to investigate potential interactions between ITGB1 and the S protein, as well as between ITGB1 and p85α. The results confirmed that ITGB1 interacted with the S1 domain of S protein (Fig. 6B and C) and p85α (Fig. 6D). Furthermore, co-transfection of plasmids expressing SADS-CoV S, ITGB1, and p85α demonstrated an interaction among all three proteins (Fig. 6E). These results suggested that ITGB1 may be the core protein linking the S protein to p85α.

To further clarify the role of ITGB1 in the activation of the PI3K/AKT/AMPK pathway following SADS-CoV infection, siRNA targeting ITGB1 was utilized. Western blot showed that knockdown of ITGB1 reduced phosphorylation of AKT at Ser473 and AMPK at Ser485 without affecting total levels (Fig. 5F and G). Additionally, downregulation of SADS-CoV N protein (Fig. 6F) and a marked decrease in viral titer were observed (Fig. 6H). These results indicate that ITGB1 interference hindered SADS-CoV-induced cholesterol synthesis and viral replication. Furthermore, IPI-2I and Vero E6 cells were treated with an Ab targeting ITGB1 to examine the effect on the PI3K/AKT/AMPK pathway post-infection. Cell viability analysis indicated that the ITGB1 Ab (20 µg/mL) showed no cytotoxicity to IPI-2I and Vero E6 cells (Fig. 6I). Levels of p-AKT, AKT p-AMPK, and AMPK in SADS-CoV infection treated with ITGB1 Ab were then assessed. A prominent reduction in phosphorylation of AKT at Ser473 and AMPK at Ser485 was observed in both cell lines (Fig. 6J and K), while total protein levels remained unchanged. In addition, downregulation of the SADS-CoV N protein (Fig. 6J) and viral titer (Fig. 6L) was also observed. These findings suggested that ITGB1 mediates activation of the PI3K/AKT/AMPK pathway induced by the SADS-CoV S protein, thereby influencing both cholesterol synthesis and viral replication.

### Cholesterol facilitates SADS-CoV S protein-mediated membrane fusion

**Fig 7:**
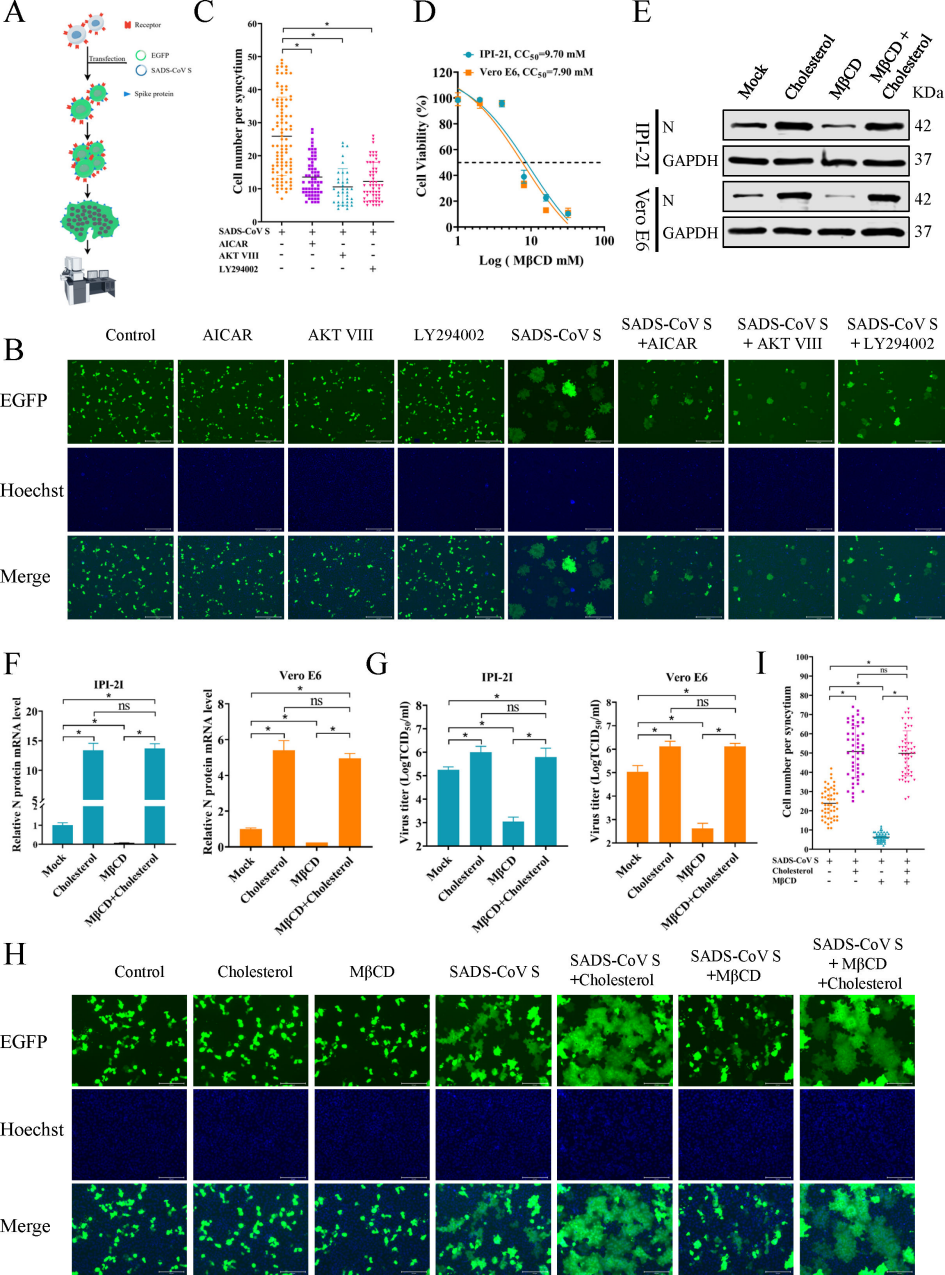
Cholesterol facilitates SADS-CoV S protein-mediated membrane fusion. (**A**) Schematic representation of the cell membrane fusion mediated by the SADS-CoV S protein. (**B**) Vero E6 cells were transfected with plasmids expressing the S protein (2 µg) and/or EGFP (0.5 µg) in 12-well plates. At 6 hpt, the medium was replaced, and AICAR (2 mM), AKT VIII (12.5 µM), and LY294002 (100 µM) were added individually. At 12 hpt, 5 µg/mL of 0.25% trypsin (without EDTA) was added, and the nuclei were stained with Hoechst 33342 for 15 min at 36 hpt. Scale bar = 125 µm. (**C**) Quantification of membrane fusion induced by SADS-CoV S protein was performed by calculating the number of cells in GFP^+^ syncytia. (**D**) Cytotoxic effects of MβCD on IPI-2I and Vero E6 cells. (E–G) The effects of MβCD and exogenous cholesterol on SADS-CoV replication. Cells were either mock-treated or pretreated with 4 mM MβCD for 1 h, followed by infection with SADS-CoV for another 1 h. Subsequently, 1 mM exogenous cholesterol and/or 4 mM MβCD were added individually or in combination, and cell samples were collected at 36 hpi. SADS-CoV N protein and mRNA levels were detected by western blot (**E**) and qRT-PCR (**F**). TCID_50_ assays were utilized to determine viral titers (**G**). (**H, I**) Vero E6 cells were transfected with plasmids expressing the S protein (2 µg) and EGFP (0.5 µg) were transfected individually or together into Vero E6 cells in 12-well plates. At 6 hpt, the medium was replaced, and cholesterol (1 mM) and/or MβCD (4 mM) were added individually or in combination as needed. At 12 hpt, 5 µg/ml of 0.25% trypsin (without EDTA) was added, and the nuclei were stained with Hoechst 33342 for 15 min at 36 hpt. Scale bar = 125 µm (**H**). Quantification of membrane fusion induced by SADS-CoV S protein was performed by calculating the number of cells in GFP^+^ syncytia (**I**). The results are reported as the means and SD (error bars) of three independent experiments (*P < 0.05).

Previous studies have shown that SARS-CoV-2 requires cholesterol for viral entry and pathological syncytium formation (21). The coronavirus S protein facilitates viral entry by binding to cellular receptors and promoting the fusion of the viral envelope with the host cell membrane (17, 47, 48). Apart from virus-mediated fusion, the S protein located on the plasma membrane can induce the formation of receptor-dependent syncytia (19). To evaluate the impact of cholesterol on syncytium formation mediated by the SADS-CoV S protein, drugs (AICAR, AKT VIII, and LY294002) were utilized to inhibit cellular cholesterol synthesis. A cell membrane fusion system was established in Vero E6 cells expressing both EGFP and SADS-CoV S proteins (Fig. 7F), as previously described (27). Vero E6 cells were transfected with plasmids encoding SADS-CoV S and EGFP, and then treated with AICAR, AKT VIII, and LY294002 at 6 hpt. Trypsin was added at 12 hpt, and the nuclei were stained with Hoechst 33342 at 36 hpt. The results showed that the three drugs significantly inhibited membrane fusion (Fig. 7B and C), indicating that interference with cellular cholesterol synthesis inhibited SADS-CoV S protein-mediated membrane fusion.

To more directly assess the role of cholesterol in SADS-CoV replication, MβCD was utilized to deplete cholesterol from the plasma membrane or supplementation with exogenous cholesterol. Cell viability assay showed that MβCD at 4 mM had no cytotoxic effects on either cell line (Fig. 7D). Cells were treated with either cholesterol or MβCD, and subsequent western blot (Fig. 7E) and qRT-PCR (Fig. 7F) analyses showed that cholesterol obviously increased SADS-CoV N protein and mRNA levels compared to mock-treated cells, while MβCD decreased expression levels. Interestingly, MβCD treatment followed by cholesterol supplementation restored N protein and mRNA levels to those of cells treated solely with cholesterol. This observation was corroborated by the TCID_50_ assay (Fig. 7G). These findings suggested that increasing cholesterol in the plasma membrane promoted replication of SADS-CoV. Subsequently, we examined the effects of cholesterol and MβCD on membrane fusion mediated by the SADS-CoV S protein. The results indicated that cholesterol significantly enhanced cell membrane fusion, while being inhibited by MβCD (Fig. 7H and I). Notably, pre-treatment with MβCD followed by supplementation also facilitated membrane fusion. Taken together, these findings demonstrated that cholesterol facilitated membrane fusion mediated by the SADS-CoV S protein to promote syncytium formation and viral replication.

## DISCUSSION

**Fig 8:**
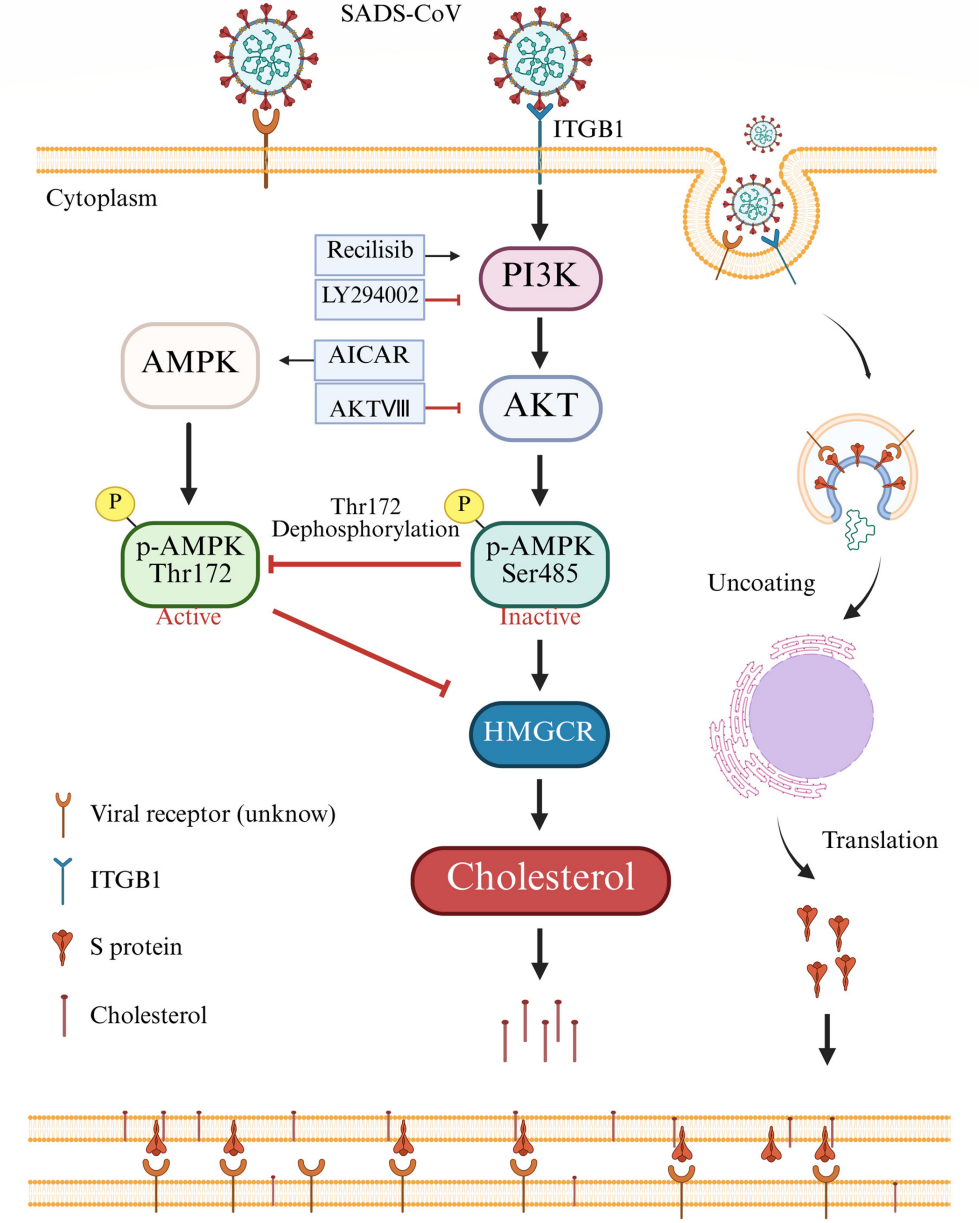
Schematic representation of SADS-CoV regulating cholesterol synthesis to favor self-replication.

Cholesterol plays a crucial role in various stages of viral invasion, translation, replication, and pathological syncytium formation, while disruption to cholesterol homeostasis impairs several critical processes during viral infection (10, 21, 49). Although multiple studies have explored the role of cholesterol in viral infection, the effects of SADS-CoV infection on cellular cholesterol and the role of cholesterol in the replication of SADS-CoV remain unclear. The results of the present study demonstrated that the SADS-CoV S protein activated the PI3K/AKT/AMPK signaling pathway via ITGB1, leading to the accumulation of cellular cholesterol, which enhanced syncytium formation and promoted viral replication (Fig. 8).

Viruses, such as HIV-1 (50), PRRSV (15), and DENV (14), have the ability to replicate by modulating cellular cholesterol biosynthesis. Likewise, SADS-CoV enhances the accumulation of cellular cholesterol. Interestingly, SARS-CoV-2 infection negatively downregulates the cholesterol synthesis pathway, while amlodipine, a calcium-channel antagonist, blocks SARS-CoV-2 infection by upregulating cholesterol synthesis (51, 52). This suggests that, despite both SADS-CoV and SARS-CoV-2 belonging to the coronavirus family, these viruses exhibit significant differences in the regulation of cellular cholesterol. Therefore, it is essential to investigate the regulation of cellular cholesterol during SADS-CoV infection and the potential relationship with viral replication mechanisms.

HMGCR activity is primarily regulated by upstream molecules PP2A and AMPK. AMPK inhibits HMGCR activity through phosphorylation, which reduces cholesterol synthesis, whereas PP2A enhances HMGCR activity through dephosphorylation, thereby promoting cholesterol synthesis (28, 29). Certain viruses can exploit the functions of AMPK, which serves as an important metabolic regulator, to manipulate cellular metabolic processes, including autophagy, fatty acid metabolism, and glucose metabolism (53). AMPK activity is regulated by the phosphorylation of the α subunit Thr172, while dephosphorylated AMPK is inactive (30). By inhibiting AMPK activity, DENV (14), HCV (34), and respiratory syncytial virus (54) create a host lipid environment that is conducive to their replication. Similar to previous reports, we found that increased HMGCR activity in SADS-CoV-infected cells occurred through inhibition of AMPK, with no influence by PP2A. Interestingly, HMGCR activity began to increase at 6 hpi and was notably enhanced by 12 hpi. Concurrently, AMPK activity did not significantly decrease at 12 hpi, but exhibited a marked decline at 24 hpi. Although HMGCR activity is theoretically expected to align with AMPK activity, our observations indicated that HMGCR upregulation occurred prior to changes in AMPK. This asynchronous phenomenon suggests that AMPK may be partially suppressed during the early stages of viral infection (14), even though AMPK activity did not immediately decrease. This subtle inhibition of AMPK may be sufficient to affect the phosphorylation state of HMGCR, thereby contributing to its dephosphorylation. Taken together, these findings indicated that SADS-CoV reduced AMPK activity, leading to less effective phosphorylation inhibition of HMGCR, which increased HMGCR activity and ultimately elevated cholesterol synthesis. Additionally, treatment with the AMPK activator AICAR indicated that increased AMPK activity inhibited HMGCR, leading to a reduction in cholesterol accumulation. In concordance, AICAR-activated AMPK exhibited significant antiviral effects, providing a potential antiviral target for SADS-CoV replication.

AKT is a crucial signal transduction protein that primarily regulates cell proliferation, survival, migration, and metabolism through phosphorylation of various substrates (55). Reportedly, AKT inhibits AMPK via phosphorylation of Ser485 of the AMPKα subunit, which affects the phosphorylation status of the key site Thr172 (31–33, 56). Some viruses exploit this pathway to promote self-replication. For instance, HCV promotes phosphorylation of Ser485 of the AMPK α subunit by phosphorylating AKT at Ser473, resulting in inhibition of AMPK activity (34). Respiratory syncytial virus suppresses AMPK activity in an AKT-dependent manner (54). In the same way, SADS-CoV enhances phosphorylation of AKT Ser473 and AMPK Ser485, suggesting that AKT activity is upregulated by SADS-CoV infection. Inhibition of AKT with AKT VIII reduced cholesterol levels and suppressed SADS-CoV replication, suggesting that AKT may be a potential antiviral target. Notably, AKT activity is primarily regulated by the upstream molecule PI3K, which both activates AKT and facilitates its localization to the cell membrane (35–37). Various viruses utilize the PI3K/AKT pathway to promote their replication (42, 43, 46, 57). Furthermore, previous studies have highlighted the critical role of the PI3K/AKT pathway in SADS-CoV infection through transcriptomic analysis and KEGG pathway enrichment (38, 45).

In the present study, modulating PI3K activity with drugs during SADS-CoV infection alters the downstream activity of AKT. We not only validated previous predictions based on gene expression data but also provided reliable experimental evidence for the role of the PI3K/AKT pathway in SADS-CoV infection. It should be noted that although both our study and that of Zeng et al. highlighted the importance of the PI3K/AKT pathway in SADS-CoV infection, the observed results differed. Here, we found that SADS-CoV activated the PI3K/AKT pathway, leading to increased AKT phosphorylation, which may support viral replication, while Zeng et al. observed a gradual decrease in AKT phosphorylation over time and found that AKT inhibition activated autophagy. These differences may be due to variations in the SADS-CoV strains. Hence, future studies should integrate time-course analysis and various intervention strategies to further explore the dynamic regulation of this pathway during SADS-CoV infection.

Earlier findings indicate that the interaction between the PI3K p85 subunit and specific viral proteins triggers the PI3K/AKT pathway (41, 44, 58, 59). The results of the current study demonstrated that SADS-CoV activated the PI3K/AKT pathway through interaction between the S protein and the PI3K p85α subunit. However, it remains unknown whether other proteins mediate this interaction. Based on our SADS-CoV host restriction factor screening results and previous studies (45, 46), ITGB1 may play a potential role between the SADS-CoV S protein and the PI3K p85α subunit. ITGB1 is widely distributed on the plasma membrane of nearly all mammalian cells and modulates various intracellular kinase pathways, including the PI3K/AKT pathway (60–63). Previous research has shown that ITGB1 activates the PI3K/AKT signaling pathway through interactions with key proteins, such as focal adhesion kinase, Src family kinases, and transforming growth factor-beta 1, thereby playing a critical role in regulating fundamental cellular processes (64). However, alternative studies have proposed that ITGB1 may also initiate the PI3K/AKT pathway via its membrane-proximal domain, independent of FAK or Src family kinases, suggesting a distinct mechanism for pathway activation (65, 66). In the present study, we demonstrate that ITGB1 acts as an adaptor protein, connecting the S protein to p85α, which subsequently activates the PI3K/AKT/AMPK pathway. Nevertheless, the exact mechanism by which ITGB1 directly engages PI3K p85α to trigger this signaling cascade requires further exploration. Collectively, these results suggested that ITGB1 not only served as a mediator of the PI3K/AKT/AMPK pathway regulated by the SADS-CoV S protein but may also act as a receptor for viral entry.

The coronavirus S protein is synthesized in the endoplasmic reticulum of host cells, matures in the Golgi apparatus, and is transported to the cell surface via vesicles, where it either embeds in the membrane or assembles with new viral particles (67). The virus interacts with surface molecules of adjacent cells via fusion proteins, leading to the formation of syncytia, which enhances viral dissemination and facilitates immune evasion (19, 20). It has been demonstrated that endogenously synthesized cholesterol is rapidly transported to the plasma membrane (68), suggesting that cholesterol induced by SADS-CoV may promote S protein-mediated membrane fusion. To mimic syncytial formation mediated by the SADS-CoV S protein, a cell membrane fusion system was established in Vero E6 cells. Treatment with three drugs that disrupted cellular cholesterol synthesis significantly inhibited SADS-CoV S protein-mediated membrane fusion. Next, the role of cholesterol in SADS-CoV replication was directly assessed by depleting cholesterol from the plasma membrane or supplementation with exogenous cholesterol. MβCD, which depletes cholesterol from the cell membrane, reduces the infectivity of various coronaviruses, including SARS-CoV-2 (69), SARS-CoV (70), infectious bronchitis virus (71), transmissible gastroenteritis virus (72), porcine deltacoronavirus (73, 74), and type I feline coronavirus (75). Similarly, MβCD significantly suppressed SADS-CoV replication, whereas exogenous cholesterol markedly enhanced viral replication, suggesting that increased cholesterol in the cell membrane favors SADS-CoV replication. Furthermore, cholesterol substantially promoted syncytium formation, while MβCD effectively inhibited this process. Also, cholesterol promoted syncytium formation during SARS-CoV-2 infection (21, 76). Nonetheless, further investigations are needed to elucidate the potential effects of cholesterol on viral translation and replication to comprehensively understand the replication mechanisms of SADS-CoV.

In conclusion, the results of the present study demonstrated that the SADS-CoV S protein upregulated intracellular cholesterol synthesis via activation of the PI3K/AKT/AMPK pathway through ITGB1. The synthesized cholesterol subsequently promotes syncytium formation mediated by the viral S protein, thereby facilitating viral replication. These findings offer novel insights into the mechanistic interplay between SADS-CoV and host cellular cholesterol metabolism, shedding light on how the virus leverages this pathway to enhance its infectivity.

## Data Availability

Data will be made available on request.

## SUPPLEMENTAL MATERIAL

Table S1 mbio.00976-25-s0001.docx Primer sequences used for qRT-PCR.

Table S2 mbio.00976-25-s0002.docx SADS-CoV proteins identified by LC-MS/MS.

ASM does not own the copyrights to Supplemental Material that may be linked to, or accessed through, an article. The authors have granted ASM a non-exclusive, world-wide license to publish the Supplemental Material files. Please contact the corresponding author directly for reuse.
