# Supplementary material for: The swine acute diarrhea syndrome coronavirus spike protein promotes syncytial formation via upregulation of cellular cholesterol synthesis
Source: mBio. 2025 Jun 30;16(8):e00976-25. doi: 10.1128/mbio.00976-25 (PMC12345205; doi:10.1128/mbio.00976-25)
Supplement: Table S1 — Primer sequences used for qRT-PCR. [file mbio.00976-25-s0001.docx]

**Table 1. Primer sequences are used for qRT-PCR in this study.**

| Target | Primer | Targeted sequences |
| --- | --- | --- |
| SADS-CoV N | 1st | CCCCTAAACCGGCTCGTAA |
|  | 2nd | CAGAATTAGGAACACGCTTCCA |
| hGAPDH | 1st | TCATGACCACAGTCCATGCC |
|  | 2nd | GGATGACCTTGCCCACAGCC |
| pGAPDH | 1st | ACCTCCACTACATGGTCTACA |
|  | 2nd | ATGACAAGCTTCCCGTTCTC |
