# Supplementary material for: The swine acute diarrhea syndrome coronavirus spike protein promotes syncytial formation via upregulation of cellular cholesterol synthesis
Source: mBio. 2025 Jun 30;16(8):e00976-25. doi: 10.1128/mbio.00976-25 (PMC12345205; doi:10.1128/mbio.00976-25)
Supplement: Table S2 — SADS-CoV proteins identified by LC-MS/MS. [file mbio.00976-25-s0002.docx]

**Table 2. SADS-CoV proteins identified by LC-MS/MS.**

| Protein name | Accession number^a^ | Mass (kDa) | Queries matched^b^ | Protein score | Peptide identified^c^ |
| --- | --- | --- | --- | --- | --- |
| Spike protein | gi\|224492556 | 126.1 | 2 | 3.7 | V.YEVASCFEASYDVLY.R  F.ISQRLDKLEADVQMD.R |
| NS3 | gi\|127236 | 25.8 | 1 | 0.004 | I.AMFVS.K |

^a^ Accession numbers according to NCBI nr database.

^b^ Number of peptides identified by LC-MS/MS is given by MaxQuant.

^c^ The peptides identified by LC-MS/MS with statistically significant ion score (confidence interval > 95%).
